# De Novo Assembly of the Peanut (*Arachis hypogaea* L.) Seed Transcriptome Revealed Candidate Unigenes for Oil Accumulation Pathways

**DOI:** 10.1371/journal.pone.0073767

**Published:** 2013-09-10

**Authors:** Dongmei Yin, Yun Wang, Xingguo Zhang, Hemin Li, Xiang Lu, Jinsong Zhang, Wanke Zhang, Shouyi Chen

**Affiliations:** 1 Henan Agricultural University, Zhengzhou, China; 2 Institute of Genetics and Developmental Biology, Chinese Academy of Sciences, Beijing, China; Key Laboratory of Horticultural Plant Biology (MOE), China

## Abstract

Peanuts are one of the most important edible oil crops in the world. In order to survey key genes controlling peanut oil accumulation, we analyzed the seed transcriptome in different developmental stages of high- and low-oil peanut varieties. About 54 million high quality clean reads were generated, which corresponded to 4.85 Gb total nucleotides. These reads were assembled into 59,236 unique sequences. Differential mRNA processing events were detected for most of the peanut Unigenes and found that 15.8% and 18.0% of the Unigenes were differentially expressed between high- and low-oil varieties at 30 DAF and 50 DAF, respectively. Over 1,500 Unigenes involved in lipid metabolism were identified, classified, and found to participate in FA synthesis and TAG assembly. There were seven possible metabolic pathways involved in the accumulation of oil during seed development. This dataset provides more sequence resource for peanut plant and will serve as the foundation to understand the mechanisms of oil accumulation in oil crops.

## Introduction

Peanuts are one of the most important edible oil crops in the world. Peanut oil has a yellowish luster, is fragrant in flavor and rich in oleic acid and linoleic acid. Peanuts have a high oil content (on average, 48-56%) compared to other oil crops. The yield of peanut oil per unit area of land is of paramount importance in supplying oil for food and non-food applications as well. Currently, there is an immediate need for the genetic enhancement and metabolic engineering of peanuts to create improved varieties for agricultural purposes including oil yield improvements. It is essential to identify candidate genes that participate in the storage lipid biosynthesis during peanut seed development, and this will serve as a foundation to create improved varieties through genetic engineering.

The quality and quantity of the storage lipids synthesized in developing seeds depends on a number of enzymes that participate in FA synthesis and TAG assembly through the Kennedy pathway [1]. Although some of the individual enzymes and genes involved in these processes are known [2–5], and changes in TAG quality or yield have been made by genetic manipulation [6–9], little is known about the overall expression and regulation proﬁles of genes involved in peanut lipid biosynthesis [10–12]. To investigate the molecular basis of storage lipid accumulation during seed development it is necessary to understand the physiological mechanisms that affect the oil content in seeds and identify the rate-limiting enzymes that regulate storage lipid biosynthesis [13,14]. During the past several years, a large number of ESTs has been generated by several cDNA sequencing studies [15–20]. Currently, about 250,000 ESTs were deposited in the NCBI Genbank. In addition to ESTs, microarray analyse has been employed for gene discovery and global gene expression profiling analyses [21]. Recently, the next generation sequence (NGS) technologies, with high throughput at a substantially reduced unit data cost, have been applied in peanuts [22–24]. There are approximately 600 genes known to be involved in lipid metabolism of *Arabidopsis* [25], but far less lipid metabolism genes are currently known from peanuts. More genes involved in lipid synthesis need to be discovered and their various functions need to be documented in peanuts to further understand the regulatory mechanisms controlling the accumulation of storage lipids.

In this study, various histological and molecular surveys were conducted from high- and low-oil peanut sublines to understand the transcriptional network that is associated with the development of lipid metabolism. Illumina HiSeq™ 2000 sequencing technology was used to profile the gene expression patterns from developing seeds and the Unigenes involved in lipid metabolism were explored to understand the major metabolic activities in peanut seeds. This dataset would provide important resource to understand the molecular basis of lipid biosynthesis and identify the rate-limiting enzymes that regulate storage lipid biosynthesis during seed development in peanuts.

## Results

### Morphology and oil accumulation of developing peanut seeds

**Figure 1:**
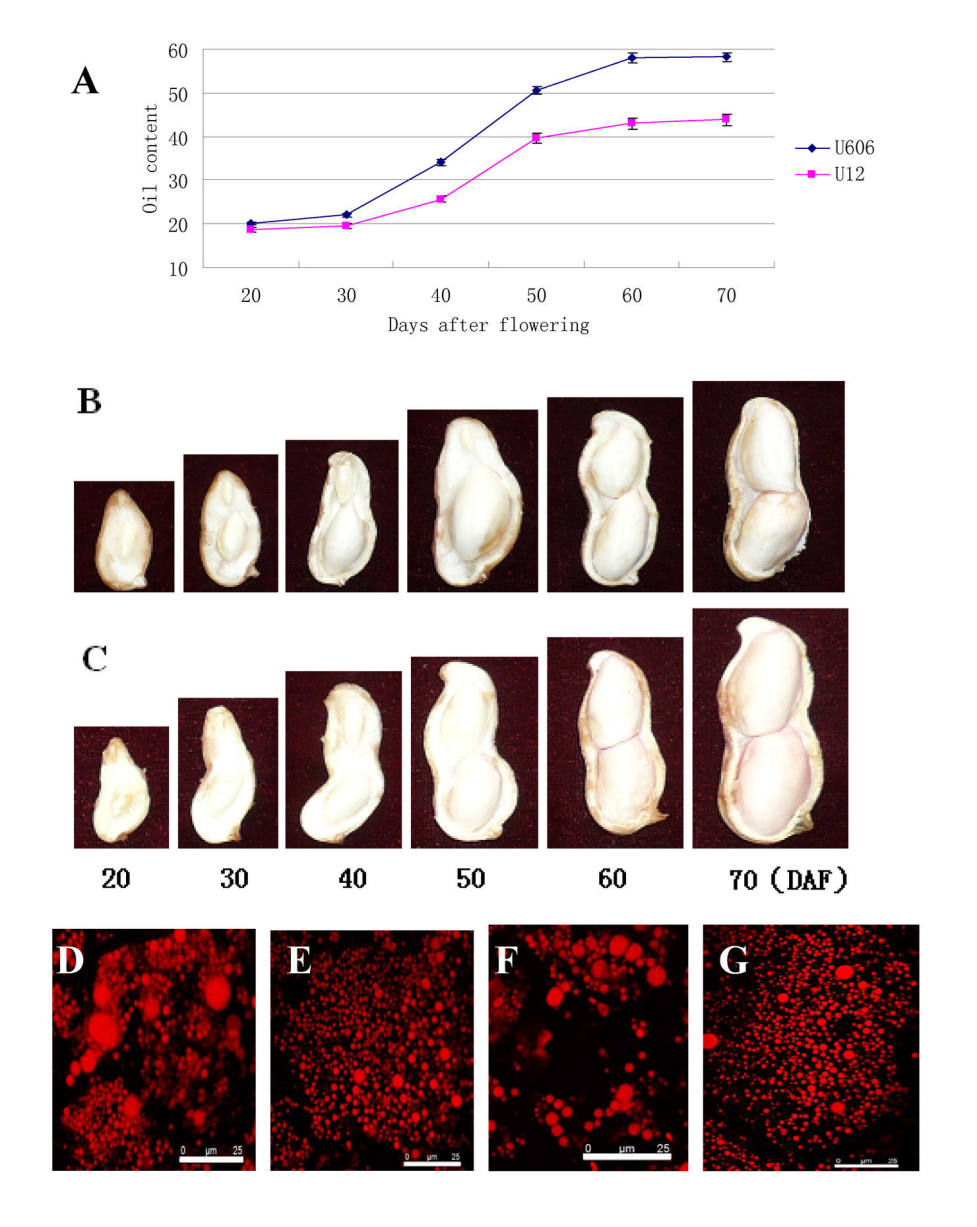
The development period of peanut seeds. (A) Time courses of oil accumulation in developing peanut seeds. Values are the average of two independent measurements, and the same seed material used. (B) The development period of U12 seeds. (C) The development period of U606 seeds. (D) and (E) A confocal section of a U606 seed at 30-40 DAF and 50-60 DAF and stained with Nile red. (F) and (G) A confocal section of a U12 seed at 30-40 DAF and 50-60 DAF and stained with Nile red. Bars = 25 µm.

The growth of peanut seeds occurred over the 70 days post fertilization in peanut plants grown in field condition. The major storage reserves accumulated from 20 DAF -70 DAF, with maximum rates of accumulation between 40 and 60 DAF. The major accumulation of storage lipids started at about 30 DAF (Figure 1A) and was indicated by an increase in total fatty acid content. Seeds at approximately 20 DAF contained 20% oil and the oil content increased to over 40% by 60 DAF. The accumulation of total lipid of U606 showed similar trend with that of U12, but the oil content of U606 seeds was increased significantly by 13% compared to that of U12 seeds at the mature stage.

It was rationalized that the mRNA changes and their regulating processes would precede the appearance of the enzymes and their products. This study focused on the period between 30 DAF and 50 DAF because this period preceded the rapid increase in storage product synthesis, and the seed biosynthetic pathways were also at their maximum activity (Figure 1B-1C). The development of oil bodies of two peanut cultivars was observed with the Nile Red confocal method (Figure 1D–1G). A few oil bodies were distributed in the center of the storage cells in the cotyledons at 30-40 DAF, and the volume and number of oil bodies per cell dramatically increased during 50-60 DAF. At 60 DAF, all oil bodies showed a globular shape, and localized in the middle of the cells. As compared with the high-oil cultivar U606, the low-oil U12 had a smaller number of uniformly sized oil bodies in storage cells. In mature seeds, the oil bodies were distributed in the central region of the storage cells and were mostly with an elliptical or irregular shape.

### De novo assembly of peanut seed transcriptomes

To profile the seed transcriptome, mRNA from seeds of cultivar U606 and U12 at 30 and 50 DAF were used to construct four cDNA libraries (named 606-3, 606-5, 12-3 and 12-5) and sequences were generated on a Illumina Genome Analyzer II platform. After removing the adaptor and low quality reads, about 54 million high quality reads were obtained from these four libraries totaling 4.8 Gb nucleotides. The average read size, Q20 percentage (sequencing error rate, 1%), and GC percentage for each library was 90 bp, >98%, and >46%, respectively. Clean reads from each library were used for assembly separately. And 58,693, 49,700, 59,819 and 52,142 Unigenes were generated from the 606-3, 606-5, 12-3 and 12-5 libraries, respectively (Figure S1). Currently, there is no complete peanut genome available, therefore these unigene data sets were further clustered to generate the final 59,236 Unigenes with a mean length of 751 nt (Table S1). Further analysis was based on this final unigene data set.

### Functional annotations of unigenes

Functional annotation provided protein functional annotation, clusters of orthologous groups (COG) annotation and gene ontology (GO) annotation of Unigenes. The unigene sequences were first aligned by blastx to protein databases including nr, Swiss-Prot, KEGG and COG (e-value < 0.00001), which retrieved proteins with the highest sequence similarity with the given Unigenes along with their protein functional annotations. Using this approach, 42,774 Unigenes (72.2% of all Unigenes) returned an above cut-off BLAST result. For Swiss-Prot analysis, up to 28,605 Unigenes (48.3% of all Unigenes) had no annotation hits.

**Figure 2:**
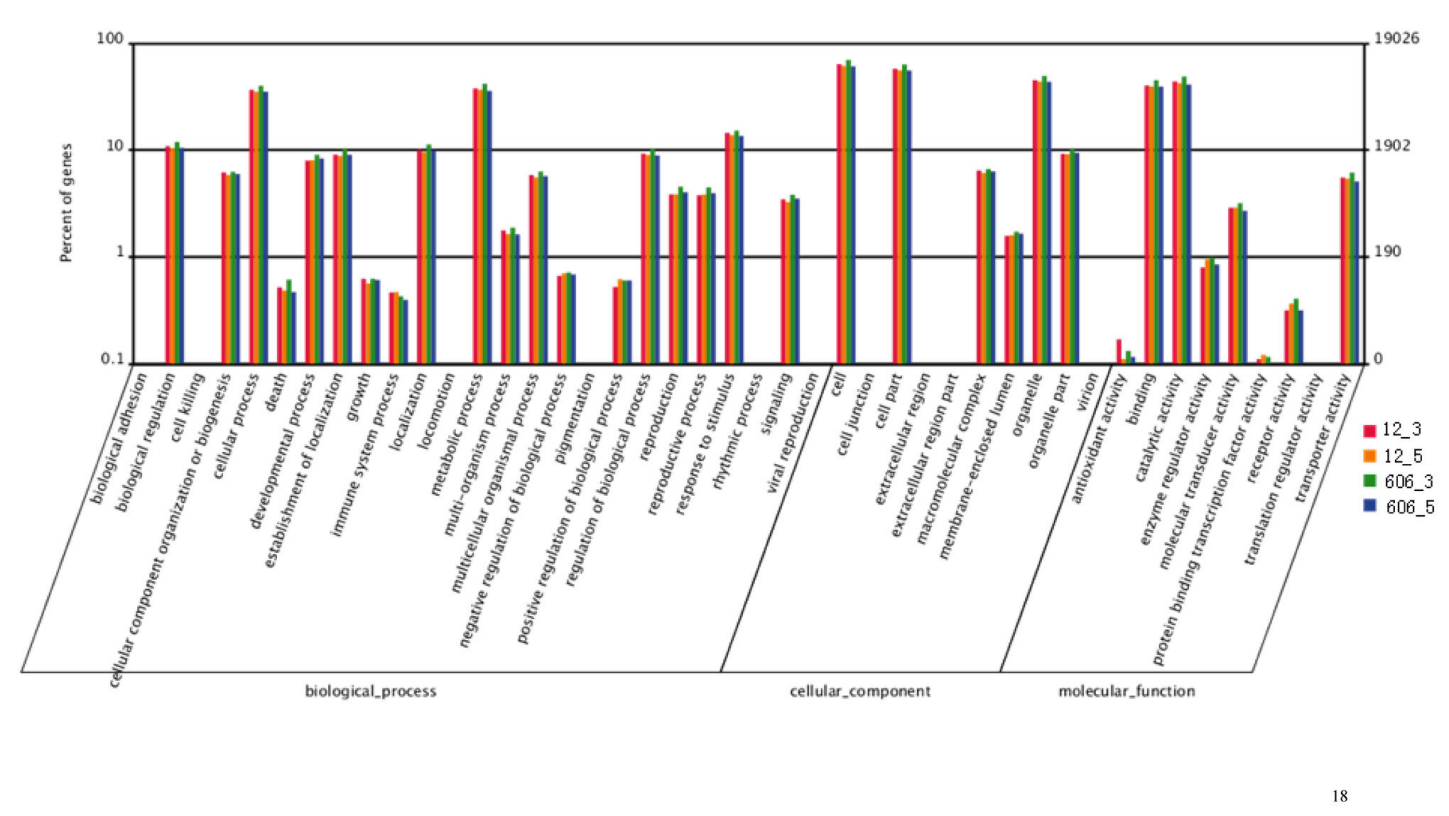
A histogram of unigene ontology classification. The results are summarized in three main categories: biological processes, cellular components and molecular functions. The right y-axis indicates the number of Unigenes in a particular category. The left y-axis indicates the percentage of a specific category of Unigenes in that main category.

The gene ontology (GO) assignments were used to categorize the functions of the predicted peanut Unigenes. Based on the sequence homologies found, 15,468 sequences were categorized into 45 functional groups (Figure 2). In each of the three main categories of the GO classification (biological processes, cellular components and molecular functions), the Unigenes attributed to “metabolic process”, “cell” and “catalytic activity” sub-categories were found in the highest percentages. A high percentage of Unigenes from the sub-categories of “cellular process”, “organelle” and “binding” were also found, but only a few Unigenes from the subsections of “biological adhesion”, “extracellular region part” and “translation regulator activity” were discovered.

**Figure 3:**
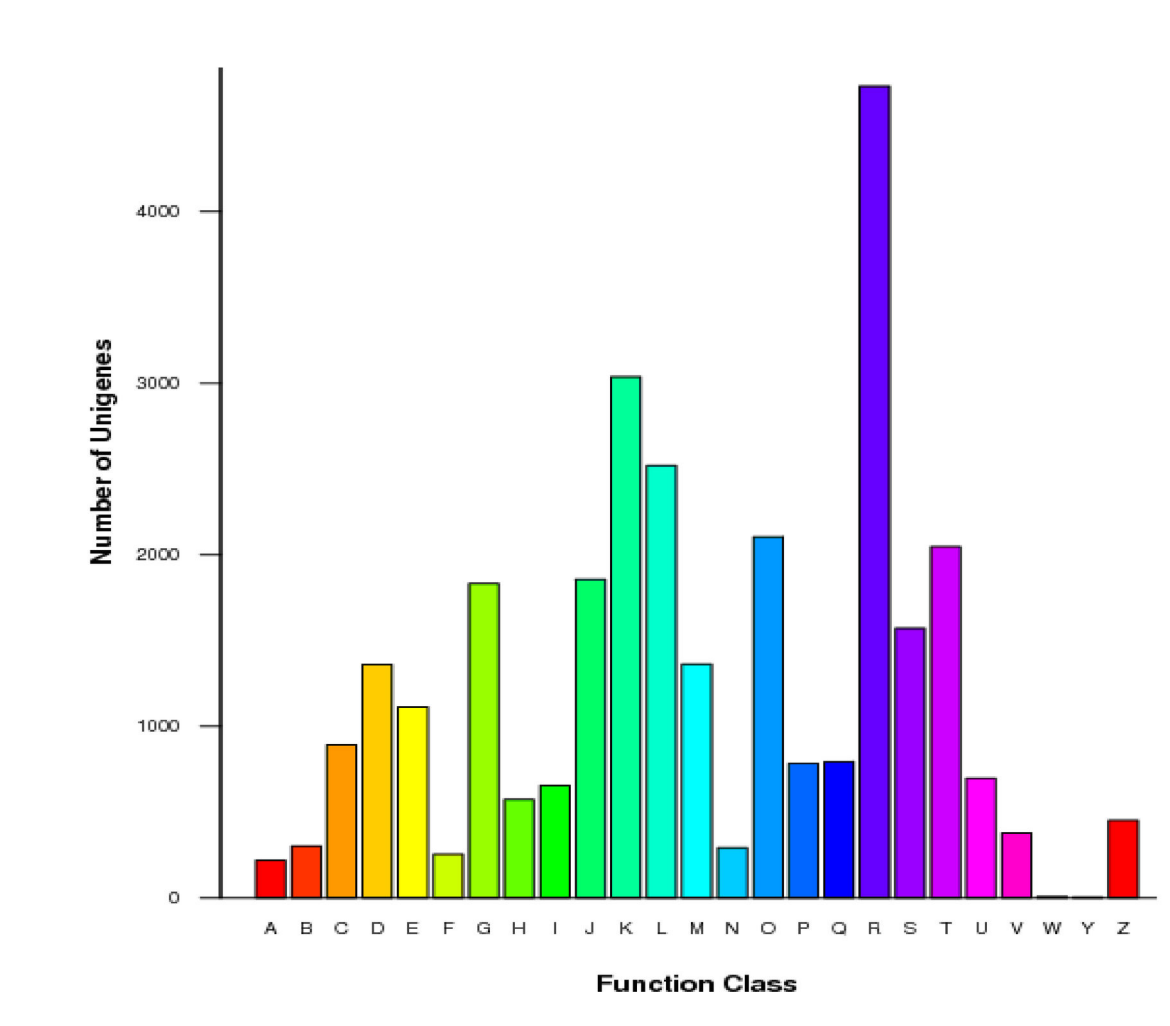
A histogram of clusters of orthologous groups (COG) classification. Out of 42,562 nr hits, 14,497 Unigenes have a COG classification within the 25 categories. (A) RNA processing and modification; (B) Chromatin structure and dynamics; (C) Energy production and conversion; (D) Cell cycle control, cell division, chromosome partitioning; (E) Amino acid transport and metabolism; (F) Nucleotide transport and metabolism; (G) Carbohydrate transport and metabolism; (H) Coenzyme transport and metabolism; (I) Lipid transport and metabolism; (J) Translation, ribosomal structure and biogenesis; (K) Transcription; (L) Replication, recombination and repair; (M) Cell wall/membrane/envelope biogenesis; (N) Cell motility; (O) Posttranslational modification, protein turnover, chaperones; (P) Inorganic ion transport and metabolism; (Q) Secondary metabolites biosynthesis, transport and catabolism; (R) General function prediction only; (S) Function unknown; (T) Signal transduction mechanisms; (U) Intracellular trafficking, secretion, and vesicular transport; (V) Defense mechanisms; (W) Extracellular structures; (Y) Nuclear structure; (Z) Cytoskeleton.

To further evaluate the completeness of our transcriptome libraries and the effectiveness of the annotation processes, we searched the previously annotated sequences for Unigenes involved in COG classifications. In total, out of the 42,562 nr hits, 14,497 sequences had a COG classification (Figure 3, Table S2). Among the 25 COG categories, the cluster for “general function prediction only” represented the largest group (4,730; 32.6%) followed by ‘‘transcription’’ (3,037; 20.9%) and “replication, recombination and repair” (2,519; 17.4%). The categories of “extracellular structures” (7; 0.05%) and “nuclear structure” (3; 0.02%) represented the smallest groups. The lipid transported metabolism group had 654 Unigenes, which accounted for 4.5% of the total Unigenes.

To identify the biological pathways that were active in the peanut plant, the 42,774 annotated sequences were mapped to the reference canonical pathways in Kyoto Encyclopedia of Genes and Genomes (KEGG). In total, 23,282 sequences were assigned to 126 KEGG pathways (Table S3). The five best represented pathways by the unique sequences were “metabolic pathways” with 5,360 members, “biosynthesis of secondary metabolites” with 2,312 members, “plant hormone signal transduction” with 1,493 members, “plant–pathogen interaction” with 1432 members and “endocytosis” with 1077 members. More than 900 members were also mapped to each of the “glycerophospholipid metabolism” (934), “spliceosome” (905 members), “RNA transport” (852) and “ether lipid metabolism” (805) subclass. These annotations provide a valuable resource for investigating specific processes, functions and pathways of the identified Unigenes.

### Gene expression profiles of developing seeds from the high- and low-oil peanut

**Figure 4:**
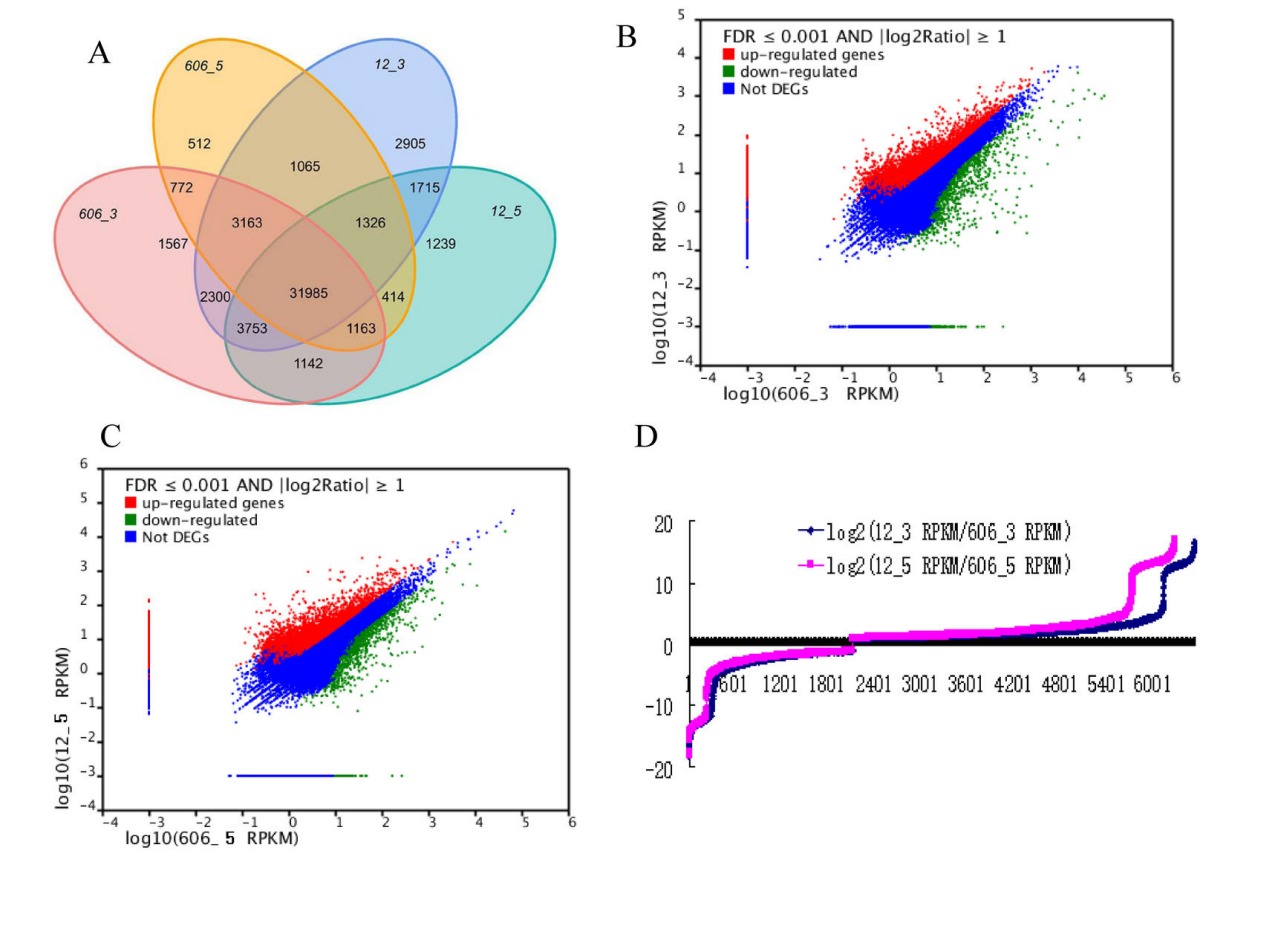
A comparison of differential unigene expression profiles among the different peanut tissues. (A) Distribution of Unigenes from mRNA-seq from the different seed tissues; (B) Expression level in 606-3vs 12-3; (C) Expression level in 606-5vs 12-5; (D) The number of upregulated or downregulated Unigenes.

To profile unigene expression for each library, clean reads from each library were mapped to 59,236 unigene. Reads mapped to multi- Unigenes were excluded. For this analysis, we artificially included Unigenes which have more than 10 mapped reads or of which more than 50% in length were covered by mapped reads. By this criterion, 55,021 out of 59,236 Unigenes were included in this analysis, among which 31,985 Unigenes were expressed in all of the four samples (Figure 4A). There were 2851 Unigenes only expressed in high-oil peanut samples (U606 libraries), and 5859 Unigenes were detected only in low-oil peanut samples (U12 libraries).

Among the 41,201 Unigenes detected between the U606-3 and U12-3 samples, we found 6,521 were differentially expressed, with more up-regulated Unigenes (4,411) than down-regulated ones (2,110) (Figure 4B, D, Table S4). And 6,265 out of 34,888 Unigenes were differentially expressed between 606–5 and 12-5 samples with more up-regulated Unigenes (4,150) than down-regulated ones (2,115) as well (Figure 4C, D, Table S5).

### Lipid pathway-specific unigenes and their functional categories

Mature peanut seeds consist of approximately 52% oil, mostly in the form of triacylglycerols (TAGs) within oil bodies. KEGG was used to automatically annotate peanut Unigenes that coded for orthologues involved in lipid metabolism. About 1,500 Unigenes were found to be involved in lipid metabolism (Table 1, Table S6). The lipid Unigenes covered most of the known cellular activities of acyl lipid metabolism, including many that were poorly characterized or recently described. There were nine cellular activities of lipid metabolism (Table S6-11). To our knowledge, this is one of the most complete and extensive efforts for lipid genes annotation of peanut plants.

**Table 1:** The main Enzymes related to oil accumulation and metabolism in peanut seeds.

**Passway**	**Symbol**	**Enzyme**	**Unigenes**
**Fatty acid biosynthesis**	DESA1	acyl-[acyl-carrier-protein] desaturase	8
	fabI	enoyl-[acyl-carrier protein] reductase I	3
	fabZ	hydroxymyristoyl ACP dehydrase	3
	FATA	fatty acyl-ACP thioesterase A	4
	FATB	fatty acyl-ACP thioesterase B	5
	accC	acetyl-CoA carboxylase, biotin carboxylase subunit	12
	FAD2	omega-6 fatty acid desaturase (delta-12 desaturase)	13
	PPT	palmitoyl-protein thioesterase	6
**Fatty acid metabolism**	PAAG	enoyl-CoA hydratase	3
	ACADM	acyl-CoA dehydrogenase	2
	DCI	methylglutaconyl-CoA hydratase	1
	ATOB	acetyl-CoA C-acetyltransferase	6
	ACAA1	acetyl-CoA acyltransferase	7
	ACSL	long-chain acyl-CoA synthetase	33
	ACOX	acyl-CoA oxidase	28
**Glycerolipid metabolism**	PDAT	phospholipid:diacylglycerol acyltransferase	10
	DGAT1	diacylglycerol O-acyltransferase	5
	GPAT3_4	glycerol-3-phosphate O-acyltransferase	8
	GPAT	glycerol-3-phosphate acyltransferase	7
	OLE	oleosin	13

**Figure 5:**
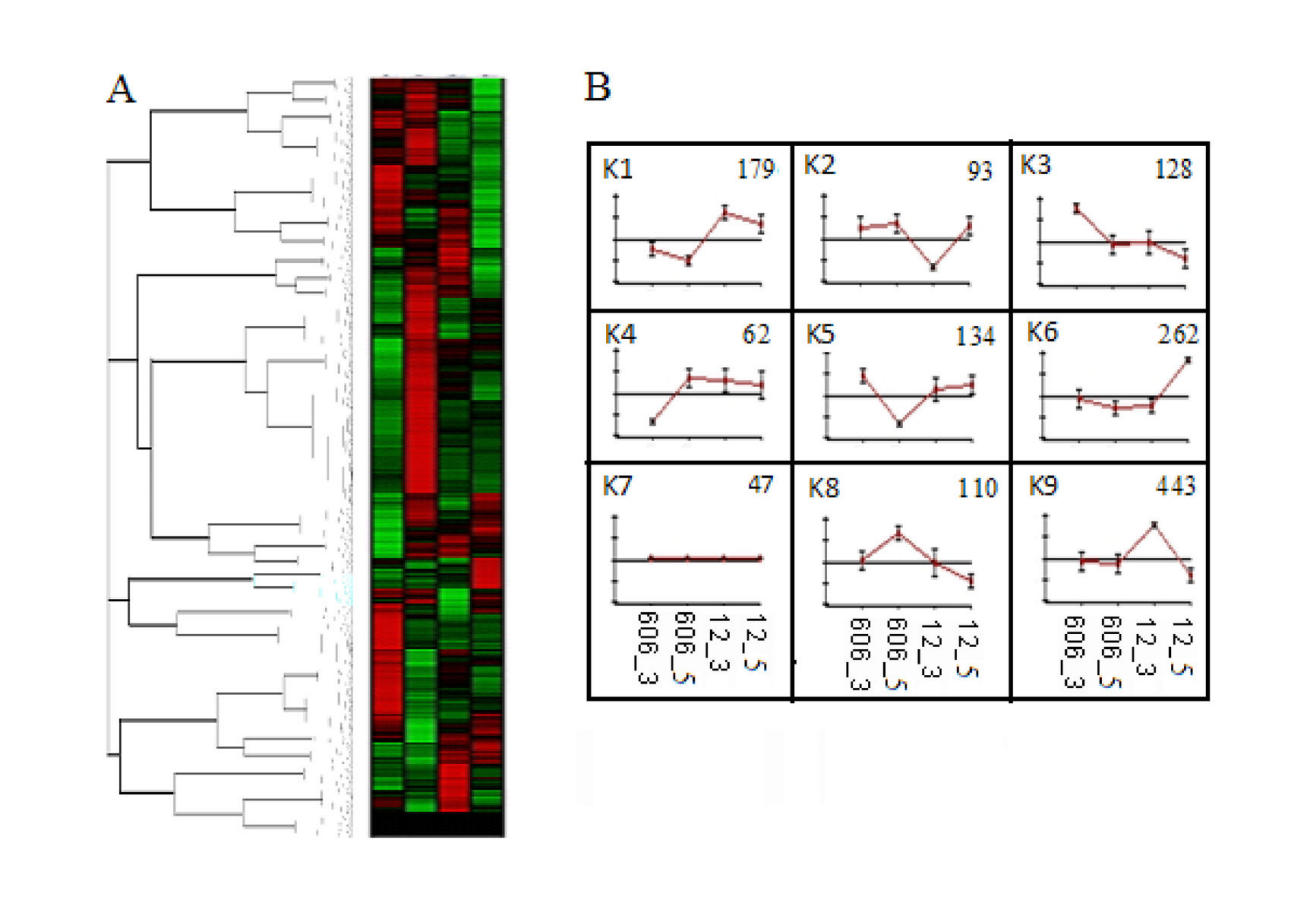
K-means clustering (A) the expression profile (B) of the Unigenes involved in lipid metabolism. The nine clusters are presented in B (K1 - K9). The error bars represent the standard deviation.

EXPANDER was used to annotate and assign Unigenes to different functional categories. The Unigenes were then grouped by developmental dynamics using the K-Means clustering algorithm and nine clusters were identified (K1-K9; Figure 5). The Unigenes that were located within the same cluster had the same or similar expression patterns during seed development. Cluster 9, covering 443 Unigenes, and cluster 1, which included 179 Unigenes, showed a major ﬂat-rise pattern, and together with cluster 6, which included 262 Unigenes, represented up-regulated clusters. Clusters 2, 3 and 8 represented down-regulated clusters, and included 93, 128 and 110 Unigenes, respectively. Cluster 7 contained 47 Unigenes and had no recognizable pattern or they have different patterns. Together, these data reveal that the major expression profiles are produced in part by highly dynamic, coordinated and localized transitions in mRNA abundance.

### Construction of metabolic pathways related to oil accumulation in peanut seeds

**Figure 6:**
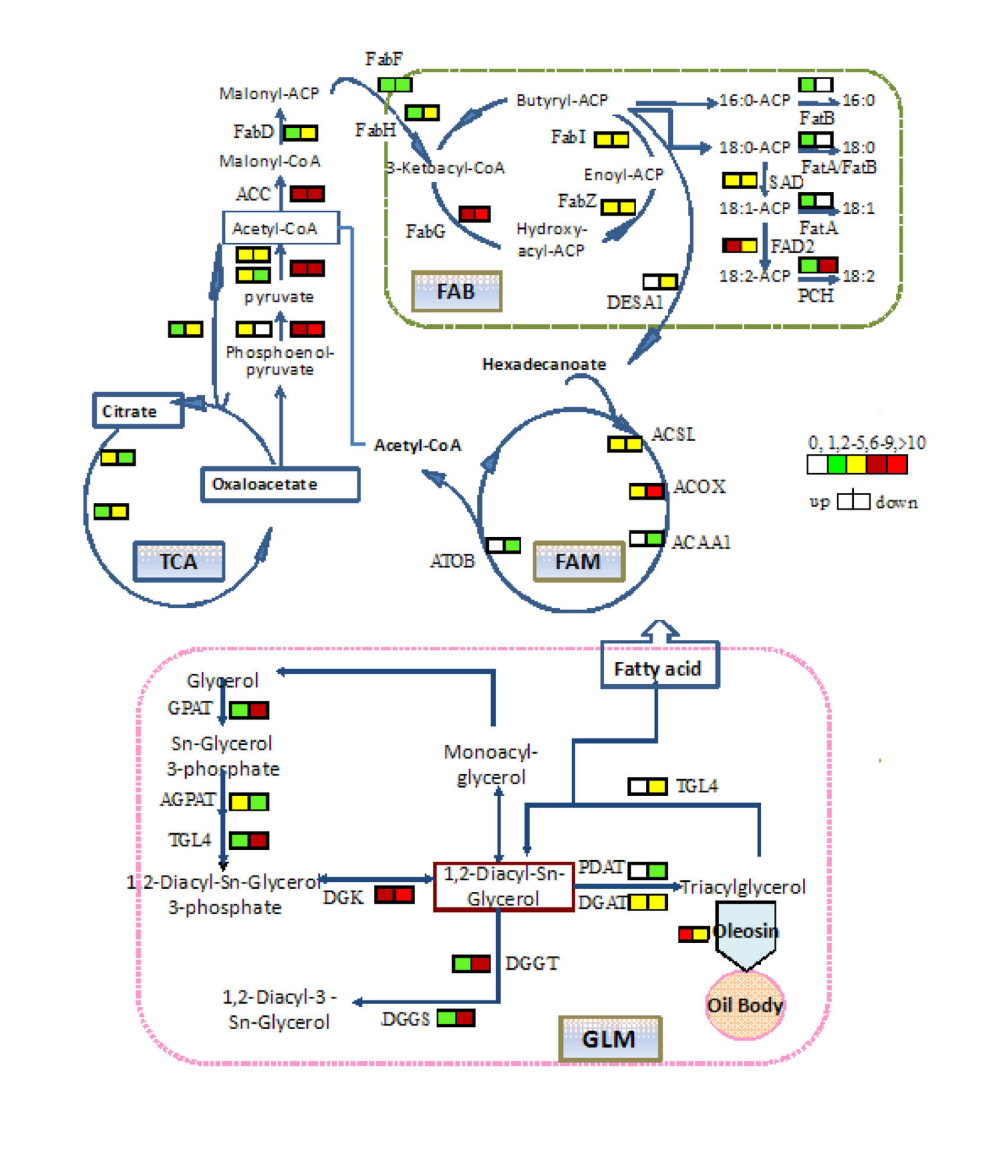
The contrapuntal patterns of unigene expression in developing peanut seeds. Peanut enzymes found in these pathways. The icons beside each enzyme name show the total number of peanut Unigenes corresponding to the enzyme in the seeds. FAB, fatty acid biosynthesis; FAM, fatty acid metabolism; GLM, glycerolipid metabolism.

KAAS [26] and KOBAS [27] were used to automatically annotate the peanut Unigenes that coded for known orthologues of plant enzymes involved in fatty acid biosynthesis, fatty acid metabolism, glycerolipid metabolism and glyceropholipid metabolism pathways. These data have been integrated and compiled to propose schematic metabolic pathways that lead to oil accumulation in peanuts seeds (Figure 6).

These results suggested that fatty acid biosynthesis, fatty acid metabolism, glycerolipid metabolism, glyceropholipid metabolism and tricarboxylic acid cycle (TCA) were all activated in the seed development process. The FAs played a fundamental role in oil mobilization and their oxidation produced 2-carbon compounds, which ultimately provided substrates for the TCA cycle. Many of the enzymes involved in fatty acid biosynthesis, fatty acid metabolism, glycerolipid metabolism and glyceropholipid metabolism pathways were up-regulated or down-regulated. ACC, FatA, FatB, SAD and FAD2 which are specific and critical enzymes in fatty acid biosynthesis were up- or down-regulated more than 10 fold (P<0.01) between the high- and low-oil peanut seeds.

## Discussion

We have assembled 59,236 expressed unigene sequences from actively growing cultivated peanut seeds using Illumina RNA-seq technology and *de novo* short read assembly. Blastx predicted 42,562 CDSs and ESTScan predicted 2,449 CDSs. There were 16,462 Unigenes that do not show homology to any genes of any plant species in UniProt using the current search methodology. Predictably, a small portion of these Unigenes were likely to represent peanut specific genes. Further studies of these Unigenes will provide new insights into peanut specific programs.

There were approximately 1,500 Unigenes involved in the lipid metabolic pathway of developing peanut seeds, and many were found to participate in fatty acid biosynthesis, fatty acid metabolism, glycerolipid metabolism, glycerophospholipid metabolism, etc. Based on our data and other extensive data, a sketch map of the metabolic processes of oil mobilization in peanuts was produced (Figure 6). Several other pathways, such as oxidation, glyoxylate cycle, glycolysis, citric acid cycle, gluconeogenesis, pentose phosphate pathway, were involved in the accumulation process. These results will aid in further understanding of the accumulation of oil in seeds.

Many enzymes, such as ACC, FAD2, DGAT, were involved in the activation to accumulate the oleosin and the lipid membrane of the oil body. Thirteen Unigenes were found to code for FAD2 catalyzing unsaturated oleoyl-ACP to linoleoyl-ACP. One unigene (unigene29528) was expressed as 1715 RPKM in 12-3, 8.5 folds to 606-3, and dramatically decreased at 50 DAF. Another unigene (unigene18953) had the same expression profile. There were five Unigenes that were expressed in higher levels in U606 than in U12. Fourteen Unigenes were found to code for 3-oxoacyl- (acyl-carrier-protein) synthase and 8 Unigenes coded for stearoyl-ACP desaturase. All of these Unigenes may have lead to a higher O/L in U12 (Table S12).

We also found five Unigenes that coded for diacylglycerol acyltransferase, which catalyzed the final step in the TAG biosynthesis pathway [6]. One of them (unigene25061) was expressed as 55 RPKM in 12-5, 2.3 folds to 606-5. Another unigene (unigene36685) was expressed as 12 RPKM in 12-3, 3 folds to 606-3. There were two Unigenes in the high-oil peanut that were expressed in higher levels than the low-oil peanut.

We found 13 Unigenes that coded for oleosin, which has an important role in the formation of oil bodies and the regulation of the size of oil bodies [28–30]. Interestingly, 10 Unigenes were more active than all of the others and all of these Unigenes were expressed in lower levels in 12-3, and in a much high level in 606-5. These Unigenes were expressed in both 606-3 and 606-5, which indicated that the high-oil peanuts had a sustained and stable expression of the team contributed to a goal. The earlier expressed Unigenes might be more important than the later expressed ones. All these Unigenes have the coordination effect, ultimately lead to the difference between high-oil and low-oil peanut.

In conclusion, we have developed a comprehensive lipid unigene resource for peanut plants that represents the developmental stages of specific seed tissues. The datasets developed in this study enhance the genomic resource database for peanuts. These resources can contribute to gene discovery and help to develop molecular markers for oil breeding studies. Additionally, the unigene set developed here might later contribute to the annotation and assembly of the whole peanut genome sequence.

## Materials and Methods

### Plant materials and tissue collection

Two peanut subline cultivars originated from the same male (Yuhua15) and female (Huayu17) parents, U606 and U12, were used. These two subline cultivars differed greatly in oil content (U606 > 58% and U12 < 45%) and fatty acid composition in mature seeds (Table S12). Days to first flower for these two cultivars are approximately 50 days. The entire growth period was approximately 120 days. Flowers at the lower position were marked and the elongating aerial pegs were later tied with colored tags. Seed tissues at specific developmental stages (20 days after flowering [DAF], 30 DAF, 40 DAF, 50 DAF, 60 DAF and 70 DAF), were harvested, quickly frozen in liquid nitrogen and stored at -80°C for RNA extraction during the 2010 growing season from May to September in Henan province.

### Lipid analysis

Seeds from each line at the six different stages were obtained from randomized growth trials. The lipid levels for each of the seeds were determined by using at least three replicates for each seed and time period. The oil content of seeds at different stages was determined by the Soxhlet extraction method. The fatty acid composition of peanut oil was analyzed by gas chromatography-mass spectrometry (Foss NIRSystems 5000).

### Microscope analyses

For laser scanning confocal microscopy, the developing seeds were cut into slices about at 50 µm thick, washed three times with a 1X PBS buffer and then infiltrated with an aqueous solution of Nile red for 15 min in the dark to visualize neutral lipids and washed again several times with 1X PBS buffer. The images were acquired with a Zeiss LSM 510 confocal laser scanning microscope.

### RNA, poly (A) RNA isolation, purification and amplification

Five seeds from the 30 DAF and 50 DAF stages were ground in liquid N_2_, and 2-5 g of the mixed sample was used for RNA isolation as described previously [31]. The total RNA was purified using the RNAeasy Mini RNA kit (Qiagen, Valencia, CA, USA). On-column DNase digestion was performed according to the manufacturer’s protocol. The Poly(A) RNA was isolated from purified total RNA using two rounds of purification with oligo d (T) beads (Dynabeads). RNA was amplified through one round using the Ribo Amp HS plus RNA amplification kit (Molecular Devices, Sunnyvale, CA, USA) following the manufacturer’s protocol. At least 100 ng of the starting total RNA was used for the amplification of each replicate. The integrity and quality of the total RNA, mRNA and amplified RNA from the tissue on the glass slides was checked by a Bioanalyzer 2100 (Agilent, Santa Clara, CA, USA). The quality cut-offs for the Bioanalyzer ananlysis is RIN (RNA Integrity Number) >7.0.

### Library preparation and Illumina sequence analyses

To construct the RNA-seq libraries, 100 ng of the fragmented ploy (A) RNA from four segments and 100 ng of the amplified RNA were used according to the manufacturer’s recommendations (Illumina). The first-strand cDNA was generated using random hexamer-primed reverse transcription. A second strand of cDNA was synthesized with the ligation of the adaptor, and the cDNA fragments of approximately 200 bp were isolated by gel electrophoresis. The cDNA fragments were amplified by 15 cycles of PCR and sequenced on the Illumina Genome Analyzer II.

### De novo unigene assembly and annotation

Reads were assembled using Trinity software with a Kmer = 25 [32]. Unigenes were defined as sequences that were without Ns and could not be extended on either end. Unigene sequences were aligned using BlastX (e-value <0.00001). Sequence orientations were determined according to the best hit in the database. Orientation and CDS of sequences which had no hits in Blast were predicted using ESTScan [33]. Original transcript sequences (5'–3') were provided if their orientations could be determined.

Unigene expression levels were calculated using the RPKM method (Reads Per kb per Million reads) [34]. The RPKM method can eliminate the influence of different gene lengths and sequencing discrepancies on the gene expression calculation. Therefore, the calculated gene expression can be directly used to compare differences in gene expression levels between samples. Functional annotations of Unigenes were performed using protein sequence similarities, the KEGG Pathway and COG and Gene Ontology (GO) databases using BlastX (e-value <0.00001). Furthermore, we obtained GO functional annotation with Nr annotation using the Blast2GO program [35]. We then used WEGO software [36] to gain the final GO functional classification for all Unigenes in order to understand the distribution of unigene functions of the peanut species. This Transcriptome Shotgun Assembly project has been deposited at DDBJ/EMBL/GenBank under the accession GAER00000000. The version described in this paper is the first version, GAER01000000.

The quantitative expression data of each unigene at various developmental stages were subjected to unigene expression clustering analysis using the k-mean clustering method provided within the software Expression Analyzer and DisplayER (EXPANDER) [37]. Input data were first standardized with mean 0 and variance 1, fixed norm and then pursued clustering with k-mean method. To view the expression patterns of clusters by a graph, the option of mean patterns with error bars operated by the EXPANDER was chosen that allows each cluster to be displayed in a separate panel with error bars representing standard deviations.

## Supporting Information

Figure S1 **The distribution of Unigenes based on length.**
(TIF)

Table S1 **The distribution of contigs and Unigenes in each library.**
(DOC)

Table S2 **A histogram of clusters of orthologous groups (COG) classification**
(DOC)

Table S3 **Unigene Metabolic Pathway Analysis.**
(DOC)

Table S4 **The list of 606-3 vs. 12-3 differentially expressed Unigenes (FDR<=0.001 and |log2Ratio| > =1).**
(XLS)

Table S5 **The list of 606-5 vs. 12-5 differentially expressed Unigenes (FDR<=0.001 and |log2Ratio| > =1).**
(XLS)

Table S6 **Enzymes related to lipid metabolism in peanut seeds.**
(XLS)

Table S7 **Peanut Unigenes in fatty acid biosynthesis and elongation pathway.**
(XLS)

Table S8 **Peanut Unigenes in fatty acid metabolism pathway.**
(XLS)

Table S9 **Peanut Unigenes in glycerolipid metabolism pathway.**
(XLS)

Table S10 **Peanut Unigenes in glycerophospholipid metabolism pathway.**
(XLS)

Table S11 **Peanut Unigenes in linoleic and alpha-linolenic acid pathway.**
(XLS)

Table S12 **Fatty acid composition of the total lipid content in mature seeds.**
(DOC)
