# Supplementary figures and images for: De Novo Assembly of the Peanut (Arachis hypogaea L.) Seed Transcriptome Revealed Candidate Unigenes for Oil Accumulation Pathways
Source: PLoS One. 2013 Sep 10;8(9):e73767. doi: 10.1371/journal.pone.0073767 (PMC3769373; doi:10.1371/journal.pone.0073767)

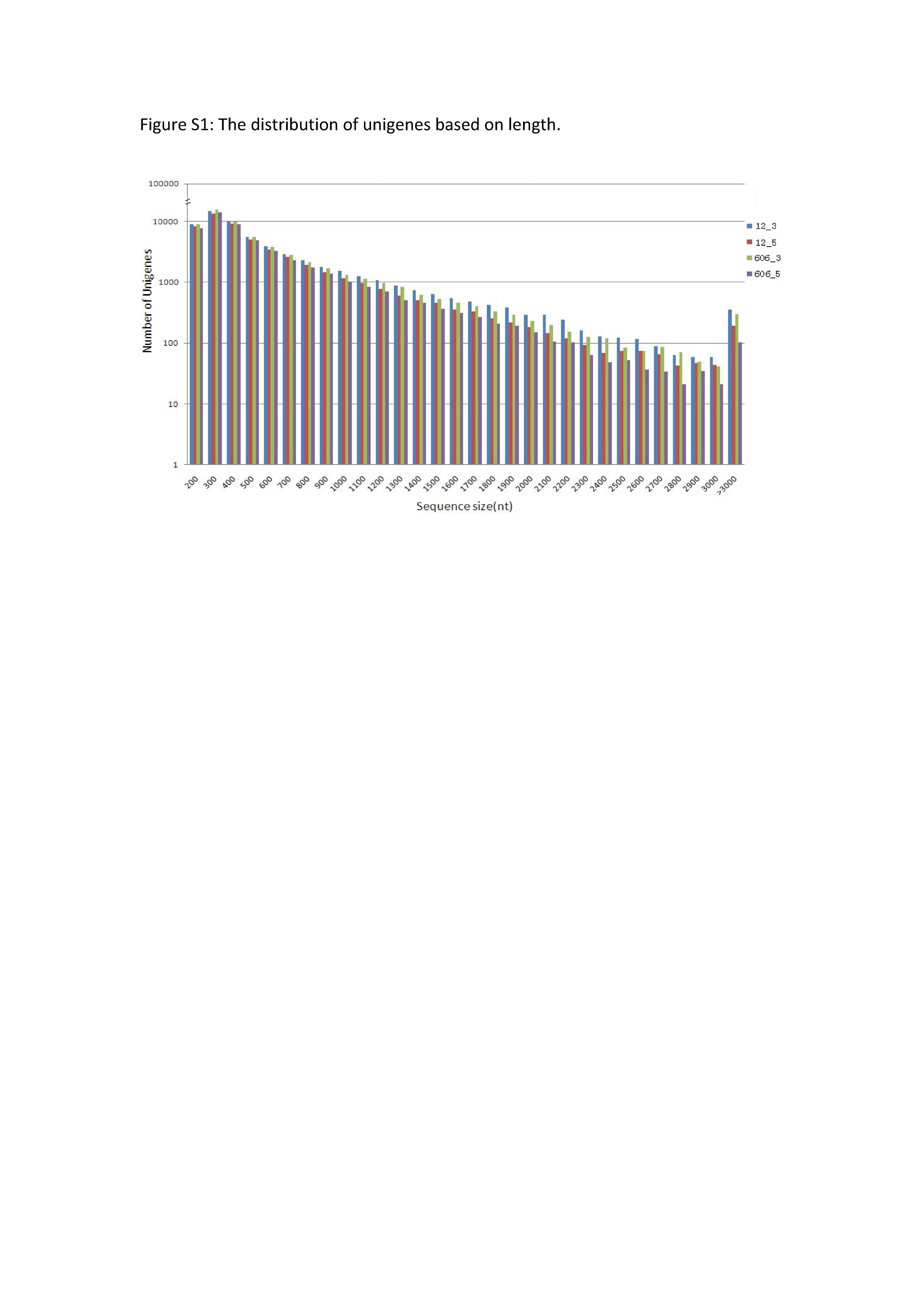

Supplement: Figure S1 — The distribution of Unigenes based on length. (TIF) [file pone.0073767.s001.tif]
