# Supplementary material for: De Novo Assembly of the Peanut (Arachis hypogaea L.) Seed Transcriptome Revealed Candidate Unigenes for Oil Accumulation Pathways
Source: PLoS One. 2013 Sep 10;8(9):e73767. doi: 10.1371/journal.pone.0073767 (PMC3769373; doi:10.1371/journal.pone.0073767)
Supplement: Table S1 — The distribution of contigs and Unigenes in each library. (DOC) [file pone.0073767.s002.doc]

Table S1. The distribution of contigs and unigenes in each library

|  | **Tissue**  **library** | **Total Number** | **Total Length (nt)** | **Mean Length (nt)** | **N50** | **Total Consensus Sequences** | **Distinct Clusters** | **Distinct Singletons** |
| --- | --- | --- | --- | --- | --- | --- | --- | --- |
| **Contig** | 606-3 | 94,239 | 30,789,225 | 327 | 482 | - | - | **-** |
|  | 606-5 | 84,357 | 25,683,798 | 304 | 425 | - | - | **-** |
|  | 12-3 | 101,120 | 33,655,683 | 333 | 522 | - | - | **-** |
|  | 12-5 | 90,264 | 27,975,304 | 310 | 451 | - | - | **-** |
| **Unigene** | 606-3 | 58,693 | 30,788,818 | 525 | 714 | 58,693 | 9,570 | **49,123** |
|  | 606-5 | 49,700 | 23,727,825 | 477 | 601 | 49,700 | 6,657 | **43,043** |
|  | 12-3 | 59,819 | 33,355,760 | 558 | 793 | 59,819 | 10,605 | **49,214** |
|  | 12-5 | 52,142 | 26,379,847 | 506 | 665 | 52,142 | 7,606 | **44,536** |
|  | All | 59,236 | 44,505,000 | 751 | 1130 | 59,236 | 41,156 | **18,080** |
