# Supplementary material for: De Novo Assembly of the Peanut (Arachis hypogaea L.) Seed Transcriptome Revealed Candidate Unigenes for Oil Accumulation Pathways
Source: PLoS One. 2013 Sep 10;8(9):e73767. doi: 10.1371/journal.pone.0073767 (PMC3769373; doi:10.1371/journal.pone.0073767)
Supplement: Table S2 — A histogram of clusters of orthologous groups (COG) classification (DOC) [file pone.0073767.s003.doc]

Table S2. A histogram of clusters of orthologous groups (COG) classification

| Code | Functional-Categories | unigenes |
| --- | --- | --- |
| A | RNA processing and modification | 220 |
| B | Chromatin structure and dynamics | 302 |
| C | Energy production and conversion | 893 |
| D | Cell cycle control, cell division, chromosome partitioning | 1358 |
| E | Amino acid transport and metabolism | 1110 |
| F | Nucleotide transport and metabolism | 254 |
| G | Carbohydrate transport and metabolism | 1832 |
| H | Coenzyme transport and metabolism | 573 |
| I | Lipid transport and metabolism | 654 |
| J | Translation, ribosomal structure and biogenesis | 1856 |
| K | Transcription | 3037 |
| L | Replication, recombination and repair | 2519 |
| M | Cell wall/membrane/envelope biogenesis | 1362 |
| N | Cell motility | 292 |
| O | Posttranslational modification, protein turnover, chaperones | 2105 |
| P | Inorganic ion transport and metabolism | 783 |
| Q | Secondary metabolites biosynthesis, transport and catabolism | 795 |
| R | General function prediction only | 4730 |
| S | Function unknown | 1571 |
| T | Signal transduction mechanisms | 2047 |
| U | Intracellular trafficking, secretion, and vesicular transport | 698 |
| V | Defense mechanisms | 377 |
| W | Extracellular structures | 7 |
| Y | Nuclear structure | 3 |
| Z | Cytoskeleton | 452 |
