# Supplementary material for: De Novo Assembly of the Peanut (Arachis hypogaea L.) Seed Transcriptome Revealed Candidate Unigenes for Oil Accumulation Pathways
Source: PLoS One. 2013 Sep 10;8(9):e73767. doi: 10.1371/journal.pone.0073767 (PMC3769373; doi:10.1371/journal.pone.0073767)
Supplement: Table S3 — Unigene Metabolic Pathway Analysis. (DOC) [file pone.0073767.s004.doc]

# Table S3. Unigene Metabolic Pathway Analysis

|  | | | | | | |
| --- | --- | --- | --- | --- | --- | --- |
| **#** | | **Pathway** | | **All unigenes with pathway annotation (23282)** | | **Pathway ID** |
| 1 | | [Metabolic pathways](../../../../E:%5C2011%E6%B5%8B%E5%BA%8F%E7%BB%93%E6%9E%9C%5Cbsi110817%5Cannotation%5CKEGG%5CAll-Unigene.fa.htm#gene1) | | 5360 (23.02%) | | ko01100 |
| 2 | | [Biosynthesis of secondary metabolites](../../../../E:%5C2011%E6%B5%8B%E5%BA%8F%E7%BB%93%E6%9E%9C%5Cbsi110817%5Cannotation%5CKEGG%5CAll-Unigene.fa.htm#gene2) | | 2312 (9.93%) | | ko01110 |
| 3 | | [Plant hormone signal transduction](../../../../E:%5C2011%E6%B5%8B%E5%BA%8F%E7%BB%93%E6%9E%9C%5Cbsi110817%5Cannotation%5CKEGG%5CAll-Unigene.fa.htm#gene3) | | 1493 (6.41%) | | ko04075 |
| 4 | | [Plant-pathogen interaction](../../../../E:%5C2011%E6%B5%8B%E5%BA%8F%E7%BB%93%E6%9E%9C%5Cbsi110817%5Cannotation%5CKEGG%5CAll-Unigene.fa.htm#gene4) | | 1432 (6.15%) | | ko04626 |
| 5 | | [Endocytosis](../../../../E:%5C2011%E6%B5%8B%E5%BA%8F%E7%BB%93%E6%9E%9C%5Cbsi110817%5Cannotation%5CKEGG%5CAll-Unigene.fa.htm#gene5) | | 1077 (4.63%) | | ko04144 |
| 6 | | [Glycerophospholipid metabolism](../../../../E:%5C2011%E6%B5%8B%E5%BA%8F%E7%BB%93%E6%9E%9C%5Cbsi110817%5Cannotation%5CKEGG%5CAll-Unigene.fa.htm#gene6) | | 934 (4.01%) | | ko00564 |
| 7 | | [Spliceosome](../../../../E:%5C2011%E6%B5%8B%E5%BA%8F%E7%BB%93%E6%9E%9C%5Cbsi110817%5Cannotation%5CKEGG%5CAll-Unigene.fa.htm#gene7) | | 905 (3.89%) | | ko03040 |
| 8 | | [RNA transport](../../../../E:%5C2011%E6%B5%8B%E5%BA%8F%E7%BB%93%E6%9E%9C%5Cbsi110817%5Cannotation%5CKEGG%5CAll-Unigene.fa.htm#gene8) | | 852 (3.66%) | | ko03013 |
| 9 | | [Ether lipid metabolism](../../../../E:%5C2011%E6%B5%8B%E5%BA%8F%E7%BB%93%E6%9E%9C%5Cbsi110817%5Cannotation%5CKEGG%5CAll-Unigene.fa.htm#gene9) | | 805 (3.46%) | | ko00565 |
| 10 | | [Purine metabolism](../../../../E:%5C2011%E6%B5%8B%E5%BA%8F%E7%BB%93%E6%9E%9C%5Cbsi110817%5Cannotation%5CKEGG%5CAll-Unigene.fa.htm#gene10) | | 633 (2.72%) | | ko00230 |
| 11 | | [Starch and sucrose metabolism](../../../../E:%5C2011%E6%B5%8B%E5%BA%8F%E7%BB%93%E6%9E%9C%5Cbsi110817%5Cannotation%5CKEGG%5CAll-Unigene.fa.htm#gene11) | | 553 (2.38%) | | ko00500 |
| 12 | | [Protein processing in endoplasmic reticulum](../../../../E:%5C2011%E6%B5%8B%E5%BA%8F%E7%BB%93%E6%9E%9C%5Cbsi110817%5Cannotation%5CKEGG%5CAll-Unigene.fa.htm#gene12) | | 551 (2.37%) | | ko04141 |
| 13 | | [Pyrimidine metabolism](../../../../E:%5C2011%E6%B5%8B%E5%BA%8F%E7%BB%93%E6%9E%9C%5Cbsi110817%5Cannotation%5CKEGG%5CAll-Unigene.fa.htm#gene13) | | 492 (2.11%) | | ko00240 |
| 14 | | [Ribosome biogenesis in eukaryotes](../../../../E:%5C2011%E6%B5%8B%E5%BA%8F%E7%BB%93%E6%9E%9C%5Cbsi110817%5Cannotation%5CKEGG%5CAll-Unigene.fa.htm#gene14) | | 465 (2%) | | ko03008 |
| 15 | | [Ribosome](../../../../E:%5C2011%E6%B5%8B%E5%BA%8F%E7%BB%93%E6%9E%9C%5Cbsi110817%5Cannotation%5CKEGG%5CAll-Unigene.fa.htm#gene15) | | 458 (1.97%) | | ko03010 |
| 16 | | [mRNA surveillance pathway](../../../../E:%5C2011%E6%B5%8B%E5%BA%8F%E7%BB%93%E6%9E%9C%5Cbsi110817%5Cannotation%5CKEGG%5CAll-Unigene.fa.htm#gene16) | | 454 (1.95%) | | ko03015 |
| 17 | | [RNA degradation](../../../../E:%5C2011%E6%B5%8B%E5%BA%8F%E7%BB%93%E6%9E%9C%5Cbsi110817%5Cannotation%5CKEGG%5CAll-Unigene.fa.htm#gene17) | | 405 (1.74%) | | ko03018 |
| 18 | | [Phenylpropanoid biosynthesis](../../../../E:%5C2011%E6%B5%8B%E5%BA%8F%E7%BB%93%E6%9E%9C%5Cbsi110817%5Cannotation%5CKEGG%5CAll-Unigene.fa.htm#gene18) | | 403 (1.73%) | | ko00940 |
| 19 | | [Ubiquitin mediated proteolysis](../../../../E:%5C2011%E6%B5%8B%E5%BA%8F%E7%BB%93%E6%9E%9C%5Cbsi110817%5Cannotation%5CKEGG%5CAll-Unigene.fa.htm#gene19) | | 398 (1.71%) | | ko04120 |
| 20 | | [Glycolysis / Gluconeogenesis](../../../../E:%5C2011%E6%B5%8B%E5%BA%8F%E7%BB%93%E6%9E%9C%5Cbsi110817%5Cannotation%5CKEGG%5CAll-Unigene.fa.htm#gene20) | | 347 (1.49%) | | ko00010 |
| 21 | | [Oxidative phosphorylation](../../../../E:%5C2011%E6%B5%8B%E5%BA%8F%E7%BB%93%E6%9E%9C%5Cbsi110817%5Cannotation%5CKEGG%5CAll-Unigene.fa.htm#gene21) | | 303 (1.3%) | | ko00190 |
| 22 | | [Amino sugar and nucleotide sugar metabolism](../../../../E:%5C2011%E6%B5%8B%E5%BA%8F%E7%BB%93%E6%9E%9C%5Cbsi110817%5Cannotation%5CKEGG%5CAll-Unigene.fa.htm#gene22) | | 281 (1.21%) | | ko00520 |
| 23 | | [Pyruvate metabolism](../../../../E:%5C2011%E6%B5%8B%E5%BA%8F%E7%BB%93%E6%9E%9C%5Cbsi110817%5Cannotation%5CKEGG%5CAll-Unigene.fa.htm#gene23) | | 266 (1.14%) | | ko00620 |
| 24 | | [Nucleotide excision repair](../../../../E:%5C2011%E6%B5%8B%E5%BA%8F%E7%BB%93%E6%9E%9C%5Cbsi110817%5Cannotation%5CKEGG%5CAll-Unigene.fa.htm#gene24) | | 252 (1.08%) | | ko03420 |
| 25 | | [Circadian rhythm - plant](../../../../E:%5C2011%E6%B5%8B%E5%BA%8F%E7%BB%93%E6%9E%9C%5Cbsi110817%5Cannotation%5CKEGG%5CAll-Unigene.fa.htm#gene25) | | 252 (1.08%) | | ko04712 |
| 26 | | [Phagosome](../../../../E:%5C2011%E6%B5%8B%E5%BA%8F%E7%BB%93%E6%9E%9C%5Cbsi110817%5Cannotation%5CKEGG%5CAll-Unigene.fa.htm#gene26) | | 251 (1.08%) | | ko04145 |
| 27 | | [Peroxisome](../../../../E:%5C2011%E6%B5%8B%E5%BA%8F%E7%BB%93%E6%9E%9C%5Cbsi110817%5Cannotation%5CKEGG%5CAll-Unigene.fa.htm#gene27) | | 242 (1.04%) | | ko04146 |
| 28 | | [RNA polymerase](../../../../E:%5C2011%E6%B5%8B%E5%BA%8F%E7%BB%93%E6%9E%9C%5Cbsi110817%5Cannotation%5CKEGG%5CAll-Unigene.fa.htm#gene28) | | 236 (1.01%) | | ko03020 |
| 29 | | [Flavonoid biosynthesis](../../../../E:%5C2011%E6%B5%8B%E5%BA%8F%E7%BB%93%E6%9E%9C%5Cbsi110817%5Cannotation%5CKEGG%5CAll-Unigene.fa.htm#gene29) | | 235 (1.01%) | | ko00941 |
| 30 | | [Stilbenoid, diarylheptanoid and gingerol biosynthesis](../../../../E:%5C2011%E6%B5%8B%E5%BA%8F%E7%BB%93%E6%9E%9C%5Cbsi110817%5Cannotation%5CKEGG%5CAll-Unigene.fa.htm#gene30) | | 228 (0.98%) | | ko00945 |
| 31 | | [Basal transcription factors](../../../../E:%5C2011%E6%B5%8B%E5%BA%8F%E7%BB%93%E6%9E%9C%5Cbsi110817%5Cannotation%5CKEGG%5CAll-Unigene.fa.htm#gene31) | | 214 (0.92%) | | ko03022 |
| 32 | | [Phosphatidylinositol signaling system](../../../../E:%5C2011%E6%B5%8B%E5%BA%8F%E7%BB%93%E6%9E%9C%5Cbsi110817%5Cannotation%5CKEGG%5CAll-Unigene.fa.htm#gene32) | | 213 (0.91%) | | ko04070 |
| 33 | | [Carbon fixation in photosynthetic organisms](../../../../E:%5C2011%E6%B5%8B%E5%BA%8F%E7%BB%93%E6%9E%9C%5Cbsi110817%5Cannotation%5CKEGG%5CAll-Unigene.fa.htm#gene33) | | 211 (0.91%) | | ko00710 |
| 34 | | [Inositol phosphate metabolism](../../../../E:%5C2011%E6%B5%8B%E5%BA%8F%E7%BB%93%E6%9E%9C%5Cbsi110817%5Cannotation%5CKEGG%5CAll-Unigene.fa.htm#gene34) | | 209 (0.9%) | | ko00562 |
| 35 | | [ABC transporters](../../../../E:%5C2011%E6%B5%8B%E5%BA%8F%E7%BB%93%E6%9E%9C%5Cbsi110817%5Cannotation%5CKEGG%5CAll-Unigene.fa.htm#gene35) | | 206 (0.88%) | | ko02010 |
| 36 | | [Base excision repair](../../../../E:%5C2011%E6%B5%8B%E5%BA%8F%E7%BB%93%E6%9E%9C%5Cbsi110817%5Cannotation%5CKEGG%5CAll-Unigene.fa.htm#gene36) | | 206 (0.88%) | | ko03410 |
| 37 | | [Homologous recombination](../../../../E:%5C2011%E6%B5%8B%E5%BA%8F%E7%BB%93%E6%9E%9C%5Cbsi110817%5Cannotation%5CKEGG%5CAll-Unigene.fa.htm#gene37) | | 204 (0.88%) | | ko03440 |
| 38 | | [Limonene and pinene degradation](../../../../E:%5C2011%E6%B5%8B%E5%BA%8F%E7%BB%93%E6%9E%9C%5Cbsi110817%5Cannotation%5CKEGG%5CAll-Unigene.fa.htm#gene38) | | 204 (0.88%) | | ko00903 |
| 39 | | [Galactose metabolism](../../../../E:%5C2011%E6%B5%8B%E5%BA%8F%E7%BB%93%E6%9E%9C%5Cbsi110817%5Cannotation%5CKEGG%5CAll-Unigene.fa.htm#gene39) | | 188 (0.81%) | | ko00052 |
| 40 | | [Cyanoamino acid metabolism](../../../../E:%5C2011%E6%B5%8B%E5%BA%8F%E7%BB%93%E6%9E%9C%5Cbsi110817%5Cannotation%5CKEGG%5CAll-Unigene.fa.htm#gene40) | | 186 (0.8%) | | ko00460 |
| 41 | | [Cysteine and methionine metabolism](../../../../E:%5C2011%E6%B5%8B%E5%BA%8F%E7%BB%93%E6%9E%9C%5Cbsi110817%5Cannotation%5CKEGG%5CAll-Unigene.fa.htm#gene41) | | 181 (0.78%) | | ko00270 |
| 42 | | [DNA replication](../../../../E:%5C2011%E6%B5%8B%E5%BA%8F%E7%BB%93%E6%9E%9C%5Cbsi110817%5Cannotation%5CKEGG%5CAll-Unigene.fa.htm#gene42) | | 174 (0.75%) | | ko03030 |
| 43 | | [Pentose and glucuronate interconversions](../../../../E:%5C2011%E6%B5%8B%E5%BA%8F%E7%BB%93%E6%9E%9C%5Cbsi110817%5Cannotation%5CKEGG%5CAll-Unigene.fa.htm#gene43) | | 172 (0.74%) | | ko00040 |
| 44 | | [Arginine and proline metabolism](../../../../E:%5C2011%E6%B5%8B%E5%BA%8F%E7%BB%93%E6%9E%9C%5Cbsi110817%5Cannotation%5CKEGG%5CAll-Unigene.fa.htm#gene44) | | 164 (0.7%) | | ko00330 |
| 45 | | [Glutathione metabolism](../../../../E:%5C2011%E6%B5%8B%E5%BA%8F%E7%BB%93%E6%9E%9C%5Cbsi110817%5Cannotation%5CKEGG%5CAll-Unigene.fa.htm#gene45) | | 164 (0.7%) | | ko00480 |
| 46 | | [Glycerolipid metabolism](../../../../E:%5C2011%E6%B5%8B%E5%BA%8F%E7%BB%93%E6%9E%9C%5Cbsi110817%5Cannotation%5CKEGG%5CAll-Unigene.fa.htm#gene46) | | 164 (0.7%) | | ko00561 |
| 47 | | [Zeatin biosynthesis](../../../../E:%5C2011%E6%B5%8B%E5%BA%8F%E7%BB%93%E6%9E%9C%5Cbsi110817%5Cannotation%5CKEGG%5CAll-Unigene.fa.htm#gene47) | | 159 (0.68%) | | ko00908 |
| 48 | | [Phenylalanine metabolism](../../../../E:%5C2011%E6%B5%8B%E5%BA%8F%E7%BB%93%E6%9E%9C%5Cbsi110817%5Cannotation%5CKEGG%5CAll-Unigene.fa.htm#gene48) | | 159 (0.68%) | | ko00360 |
| 49 | | [Alanine, aspartate and glutamate metabolism](../../../../E:%5C2011%E6%B5%8B%E5%BA%8F%E7%BB%93%E6%9E%9C%5Cbsi110817%5Cannotation%5CKEGG%5CAll-Unigene.fa.htm#gene49) | | 150 (0.64%) | | ko00250 |
| 50 | | [Aminoacyl-tRNA biosynthesis](../../../../E:%5C2011%E6%B5%8B%E5%BA%8F%E7%BB%93%E6%9E%9C%5Cbsi110817%5Cannotation%5CKEGG%5CAll-Unigene.fa.htm#gene50) | | 149 (0.64%) | | ko00970 |
| 51 | | [Carotenoid biosynthesis](../../../../E:%5C2011%E6%B5%8B%E5%BA%8F%E7%BB%93%E6%9E%9C%5Cbsi110817%5Cannotation%5CKEGG%5CAll-Unigene.fa.htm#gene51) | | 146 (0.63%) | | ko00906 |
| 52 | | [Fatty acid metabolism](../../../../E:%5C2011%E6%B5%8B%E5%BA%8F%E7%BB%93%E6%9E%9C%5Cbsi110817%5Cannotation%5CKEGG%5CAll-Unigene.fa.htm#gene52) | | 145 (0.62%) | | ko00071 |
| 53 | | [Porphyrin and chlorophyll metabolism](../../../../E:%5C2011%E6%B5%8B%E5%BA%8F%E7%BB%93%E6%9E%9C%5Cbsi110817%5Cannotation%5CKEGG%5CAll-Unigene.fa.htm#gene53) | | 141 (0.61%) | | ko00860 |
| 54 | | [Regulation of autophagy](../../../../E:%5C2011%E6%B5%8B%E5%BA%8F%E7%BB%93%E6%9E%9C%5Cbsi110817%5Cannotation%5CKEGG%5CAll-Unigene.fa.htm#gene54) | | 141 (0.61%) | | ko04140 |
| 55 | | [alpha-Linolenic acid metabolism](../../../../E:%5C2011%E6%B5%8B%E5%BA%8F%E7%BB%93%E6%9E%9C%5Cbsi110817%5Cannotation%5CKEGG%5CAll-Unigene.fa.htm#gene55) | | 138 (0.59%) | | ko00592 |
| 56 | | [Fructose and mannose metabolism](../../../../E:%5C2011%E6%B5%8B%E5%BA%8F%E7%BB%93%E6%9E%9C%5Cbsi110817%5Cannotation%5CKEGG%5CAll-Unigene.fa.htm#gene56) | | 134 (0.58%) | | ko00051 |
| 57 | | [Mismatch repair](../../../../E:%5C2011%E6%B5%8B%E5%BA%8F%E7%BB%93%E6%9E%9C%5Cbsi110817%5Cannotation%5CKEGG%5CAll-Unigene.fa.htm#gene57) | | 130 (0.56%) | | ko03430 |
| 58 | | [Tyrosine metabolism](../../../../E:%5C2011%E6%B5%8B%E5%BA%8F%E7%BB%93%E6%9E%9C%5Cbsi110817%5Cannotation%5CKEGG%5CAll-Unigene.fa.htm#gene58) | | 129 (0.55%) | | ko00350 |
| 59 | | [Citrate cycle (TCA cycle)](../../../../E:%5C2011%E6%B5%8B%E5%BA%8F%E7%BB%93%E6%9E%9C%5Cbsi110817%5Cannotation%5CKEGG%5CAll-Unigene.fa.htm#gene59) | | 127 (0.55%) | | ko00020 |
| 60 | | [Ascorbate and aldarate metabolism](../../../../E:%5C2011%E6%B5%8B%E5%BA%8F%E7%BB%93%E6%9E%9C%5Cbsi110817%5Cannotation%5CKEGG%5CAll-Unigene.fa.htm#gene60) | | 126 (0.54%) | | ko00053 |
| 61 | | [Pentose phosphate pathway](../../../../E:%5C2011%E6%B5%8B%E5%BA%8F%E7%BB%93%E6%9E%9C%5Cbsi110817%5Cannotation%5CKEGG%5CAll-Unigene.fa.htm#gene61) | | 124 (0.53%) | | ko00030 |
| 62 | | [Valine, leucine and isoleucine degradation](../../../../E:%5C2011%E6%B5%8B%E5%BA%8F%E7%BB%93%E6%9E%9C%5Cbsi110817%5Cannotation%5CKEGG%5CAll-Unigene.fa.htm#gene62) | | 123 (0.53%) | | ko00280 |
| 63 | | [Tryptophan metabolism](../../../../E:%5C2011%E6%B5%8B%E5%BA%8F%E7%BB%93%E6%9E%9C%5Cbsi110817%5Cannotation%5CKEGG%5CAll-Unigene.fa.htm#gene63) | | 121 (0.52%) | | ko00380 |
| 64 | | [Biosynthesis of unsaturated fatty acids](../../../../E:%5C2011%E6%B5%8B%E5%BA%8F%E7%BB%93%E6%9E%9C%5Cbsi110817%5Cannotation%5CKEGG%5CAll-Unigene.fa.htm#gene64) | | 116 (0.5%) | | ko01040 |
| 65 | | [Nitrogen metabolism](../../../../E:%5C2011%E6%B5%8B%E5%BA%8F%E7%BB%93%E6%9E%9C%5Cbsi110817%5Cannotation%5CKEGG%5CAll-Unigene.fa.htm#gene65) | | 113 (0.49%) | | ko00910 |
| 66 | | [Photosynthesis](../../../../E:%5C2011%E6%B5%8B%E5%BA%8F%E7%BB%93%E6%9E%9C%5Cbsi110817%5Cannotation%5CKEGG%5CAll-Unigene.fa.htm#gene66) | | 111 (0.48%) | | ko00195 |
| 67 | | [Terpenoid backbone biosynthesis](../../../../E:%5C2011%E6%B5%8B%E5%BA%8F%E7%BB%93%E6%9E%9C%5Cbsi110817%5Cannotation%5CKEGG%5CAll-Unigene.fa.htm#gene67) | | 105 (0.45%) | | ko00900 |
| 68 | | [Ubiquinone and other terpenoid-quinone biosynthesis](../../../../E:%5C2011%E6%B5%8B%E5%BA%8F%E7%BB%93%E6%9E%9C%5Cbsi110817%5Cannotation%5CKEGG%5CAll-Unigene.fa.htm#gene68) | | 105 (0.45%) | | ko00130 |
| 69 | | [SNARE interactions in vesicular transport](../../../../E:%5C2011%E6%B5%8B%E5%BA%8F%E7%BB%93%E6%9E%9C%5Cbsi110817%5Cannotation%5CKEGG%5CAll-Unigene.fa.htm#gene69) | | 101 (0.43%) | | ko04130 |
| 70 | | [Butanoate metabolism](../../../../E:%5C2011%E6%B5%8B%E5%BA%8F%E7%BB%93%E6%9E%9C%5Cbsi110817%5Cannotation%5CKEGG%5CAll-Unigene.fa.htm#gene70) | | 99 (0.43%) | | ko00650 |
| 71 | | [Glycine, serine and threonine metabolism](../../../../E:%5C2011%E6%B5%8B%E5%BA%8F%E7%BB%93%E6%9E%9C%5Cbsi110817%5Cannotation%5CKEGG%5CAll-Unigene.fa.htm#gene71) | | 98 (0.42%) | | ko00260 |
| 72 | | [Valine, leucine and isoleucine biosynthesis](../../../../E:%5C2011%E6%B5%8B%E5%BA%8F%E7%BB%93%E6%9E%9C%5Cbsi110817%5Cannotation%5CKEGG%5CAll-Unigene.fa.htm#gene72) | | 95 (0.41%) | | ko00290 |
| 73 | | [Propanoate metabolism](../../../../E:%5C2011%E6%B5%8B%E5%BA%8F%E7%BB%93%E6%9E%9C%5Cbsi110817%5Cannotation%5CKEGG%5CAll-Unigene.fa.htm#gene73) | | 92 (0.4%) | | ko00640 |
| 74 | | [Lysine degradation](../../../../E:%5C2011%E6%B5%8B%E5%BA%8F%E7%BB%93%E6%9E%9C%5Cbsi110817%5Cannotation%5CKEGG%5CAll-Unigene.fa.htm#gene74) | | 90 (0.39%) | | ko00310 |
| 75 | | [Other glycan degradation](../../../../E:%5C2011%E6%B5%8B%E5%BA%8F%E7%BB%93%E6%9E%9C%5Cbsi110817%5Cannotation%5CKEGG%5CAll-Unigene.fa.htm#gene75) | | 89 (0.38%) | | ko00511 |
| 76 | | [Phenylalanine, tyrosine and tryptophan biosynthesis](../../../../E:%5C2011%E6%B5%8B%E5%BA%8F%E7%BB%93%E6%9E%9C%5Cbsi110817%5Cannotation%5CKEGG%5CAll-Unigene.fa.htm#gene76) | | 89 (0.38%) | | ko00400 |
| 77 | | [Sphingolipid metabolism](../../../../E:%5C2011%E6%B5%8B%E5%BA%8F%E7%BB%93%E6%9E%9C%5Cbsi110817%5Cannotation%5CKEGG%5CAll-Unigene.fa.htm#gene77) | | 88 (0.38%) | | ko00600 |
| 78 | | [Natural killer cell mediated cytotoxicity](../../../../E:%5C2011%E6%B5%8B%E5%BA%8F%E7%BB%93%E6%9E%9C%5Cbsi110817%5Cannotation%5CKEGG%5CAll-Unigene.fa.htm#gene78) | | 87 (0.37%) | | ko04650 |
| 79 | | [Proteasome](../../../../E:%5C2011%E6%B5%8B%E5%BA%8F%E7%BB%93%E6%9E%9C%5Cbsi110817%5Cannotation%5CKEGG%5CAll-Unigene.fa.htm#gene79) | | 86 (0.37%) | | ko03050 |
| 80 | | [Fatty acid biosynthesis](../../../../E:%5C2011%E6%B5%8B%E5%BA%8F%E7%BB%93%E6%9E%9C%5Cbsi110817%5Cannotation%5CKEGG%5CAll-Unigene.fa.htm#gene80) | | 85 (0.37%) | | ko00061 |
| 81 | | [Protein export](../../../../E:%5C2011%E6%B5%8B%E5%BA%8F%E7%BB%93%E6%9E%9C%5Cbsi110817%5Cannotation%5CKEGG%5CAll-Unigene.fa.htm#gene81) | | 84 (0.36%) | | ko03060 |
| 82 | | [Glycosaminoglycan degradation](../../../../E:%5C2011%E6%B5%8B%E5%BA%8F%E7%BB%93%E6%9E%9C%5Cbsi110817%5Cannotation%5CKEGG%5CAll-Unigene.fa.htm#gene82) | | 84 (0.36%) | | ko00531 |
| 83 | | [Glyoxylate and dicarboxylate metabolism](../../../../E:%5C2011%E6%B5%8B%E5%BA%8F%E7%BB%93%E6%9E%9C%5Cbsi110817%5Cannotation%5CKEGG%5CAll-Unigene.fa.htm#gene83) | | 81 (0.35%) | | ko00630 |
| 84 | | [Sulfur metabolism](../../../../E:%5C2011%E6%B5%8B%E5%BA%8F%E7%BB%93%E6%9E%9C%5Cbsi110817%5Cannotation%5CKEGG%5CAll-Unigene.fa.htm#gene84) | | 80 (0.34%) | | ko00920 |
| 85 | | [beta-Alanine metabolism](../../../../E:%5C2011%E6%B5%8B%E5%BA%8F%E7%BB%93%E6%9E%9C%5Cbsi110817%5Cannotation%5CKEGG%5CAll-Unigene.fa.htm#gene85) | | 79 (0.34%) | | ko00410 |
| 86 | | [N-Glycan biosynthesis](../../../../E:%5C2011%E6%B5%8B%E5%BA%8F%E7%BB%93%E6%9E%9C%5Cbsi110817%5Cannotation%5CKEGG%5CAll-Unigene.fa.htm#gene86) | | 76 (0.33%) | | ko00510 |
| 87 | | [Steroid biosynthesis](../../../../E:%5C2011%E6%B5%8B%E5%BA%8F%E7%BB%93%E6%9E%9C%5Cbsi110817%5Cannotation%5CKEGG%5CAll-Unigene.fa.htm#gene87) | | 75 (0.32%) | | ko00100 |
| 88 | | [Pantothenate and CoA biosynthesis](../../../../E:%5C2011%E6%B5%8B%E5%BA%8F%E7%BB%93%E6%9E%9C%5Cbsi110817%5Cannotation%5CKEGG%5CAll-Unigene.fa.htm#gene88) | | 74 (0.32%) | | ko00770 |
| 89 | | [Flavone and flavonol biosynthesis](../../../../E:%5C2011%E6%B5%8B%E5%BA%8F%E7%BB%93%E6%9E%9C%5Cbsi110817%5Cannotation%5CKEGG%5CAll-Unigene.fa.htm#gene89) | | 73 (0.31%) | | ko00944 |
| 90 | [Glycosylphosphatidylinositol(GPI)-anchor biosynthesis](../../../../E:%5C2011%E6%B5%8B%E5%BA%8F%E7%BB%93%E6%9E%9C%5Cbsi110817%5Cannotation%5CKEGG%5CAll-Unigene.fa.htm#gene90) | | 72 (0.31%) | | ko00563 | |
| 91 | [Diterpenoid biosynthesis](../../../../E:%5C2011%E6%B5%8B%E5%BA%8F%E7%BB%93%E6%9E%9C%5Cbsi110817%5Cannotation%5CKEGG%5CAll-Unigene.fa.htm#gene91) | | 71 (0.3%) | | ko00904 | |
| 92 | [Histidine metabolism](../../../../E:%5C2011%E6%B5%8B%E5%BA%8F%E7%BB%93%E6%9E%9C%5Cbsi110817%5Cannotation%5CKEGG%5CAll-Unigene.fa.htm#gene92) | | 64 (0.27%) | | ko00340 | |
| 93 | [Linoleic acid metabolism](../../../../E:%5C2011%E6%B5%8B%E5%BA%8F%E7%BB%93%E6%9E%9C%5Cbsi110817%5Cannotation%5CKEGG%5CAll-Unigene.fa.htm#gene93) | | 61 (0.26%) | | ko00591 | |
| 94 | [Glycosphingolipid biosynthesis - ganglio series](../../../../E:%5C2011%E6%B5%8B%E5%BA%8F%E7%BB%93%E6%9E%9C%5Cbsi110817%5Cannotation%5CKEGG%5CAll-Unigene.fa.htm#gene94) | | 54 (0.23%) | | ko00604 | |
| 95 | [Benzoxazinoid biosynthesis](../../../../E:%5C2011%E6%B5%8B%E5%BA%8F%E7%BB%93%E6%9E%9C%5Cbsi110817%5Cannotation%5CKEGG%5CAll-Unigene.fa.htm#gene95) | | 54 (0.23%) | | ko00402 | |
| 96 | [Non-homologous end-joining](../../../../E:%5C2011%E6%B5%8B%E5%BA%8F%E7%BB%93%E6%9E%9C%5Cbsi110817%5Cannotation%5CKEGG%5CAll-Unigene.fa.htm#gene96) | | 47 (0.2%) | | ko03450 | |
| 97 | [Folate biosynthesis](../../../../E:%5C2011%E6%B5%8B%E5%BA%8F%E7%BB%93%E6%9E%9C%5Cbsi110817%5Cannotation%5CKEGG%5CAll-Unigene.fa.htm#gene97) | | 46 (0.2%) | | ko00790 | |
| 98 | [One carbon pool by folate](../../../../E:%5C2011%E6%B5%8B%E5%BA%8F%E7%BB%93%E6%9E%9C%5Cbsi110817%5Cannotation%5CKEGG%5CAll-Unigene.fa.htm#gene98) | | 46 (0.2%) | | ko00670 | |
| 99 | [Selenocompound metabolism](../../../../E:%5C2011%E6%B5%8B%E5%BA%8F%E7%BB%93%E6%9E%9C%5Cbsi110817%5Cannotation%5CKEGG%5CAll-Unigene.fa.htm#gene99) | | 42 (0.18%) | | ko00450 | |
| 100 | [Circadian rhythm - mammal](../../../../E:%5C2011%E6%B5%8B%E5%BA%8F%E7%BB%93%E6%9E%9C%5Cbsi110817%5Cannotation%5CKEGG%5CAll-Unigene.fa.htm#gene100) | | 41 (0.18%) | | ko04710 | |
| 101 | [Nicotinate and nicotinamide metabolism](../../../../E:%5C2011%E6%B5%8B%E5%BA%8F%E7%BB%93%E6%9E%9C%5Cbsi110817%5Cannotation%5CKEGG%5CAll-Unigene.fa.htm#gene101) | | 40 (0.17%) | | ko00760 | |
| 102 | [Isoquinoline alkaloid biosynthesis](../../../../E:%5C2011%E6%B5%8B%E5%BA%8F%E7%BB%93%E6%9E%9C%5Cbsi110817%5Cannotation%5CKEGG%5CAll-Unigene.fa.htm#gene102) | | 38 (0.16%) | | ko00950 | |
| 103 | [Riboflavin metabolism](../../../../E:%5C2011%E6%B5%8B%E5%BA%8F%E7%BB%93%E6%9E%9C%5Cbsi110817%5Cannotation%5CKEGG%5CAll-Unigene.fa.htm#gene103) | | 36 (0.15%) | | ko00740 | |
| 104 | [Tropane, piperidine and pyridine alkaloid biosynthesis](../../../../E:%5C2011%E6%B5%8B%E5%BA%8F%E7%BB%93%E6%9E%9C%5Cbsi110817%5Cannotation%5CKEGG%5CAll-Unigene.fa.htm#gene104) | | 35 (0.15%) | | ko00960 | |
| 105 | [Photosynthesis - antenna proteins](../../../../E:%5C2011%E6%B5%8B%E5%BA%8F%E7%BB%93%E6%9E%9C%5Cbsi110817%5Cannotation%5CKEGG%5CAll-Unigene.fa.htm#gene105) | | 34 (0.15%) | | ko00196 | |
| 106 | [Lysine biosynthesis](../../../../E:%5C2011%E6%B5%8B%E5%BA%8F%E7%BB%93%E6%9E%9C%5Cbsi110817%5Cannotation%5CKEGG%5CAll-Unigene.fa.htm#gene106) | | 34 (0.15%) | | ko00300 | |
| 107 | [Glucosinolate biosynthesis](../../../../E:%5C2011%E6%B5%8B%E5%BA%8F%E7%BB%93%E6%9E%9C%5Cbsi110817%5Cannotation%5CKEGG%5CAll-Unigene.fa.htm#gene107) | | 33 (0.14%) | | ko00966 | |
| 108 | [Taurine and hypotaurine metabolism](../../../../E:%5C2011%E6%B5%8B%E5%BA%8F%E7%BB%93%E6%9E%9C%5Cbsi110817%5Cannotation%5CKEGG%5CAll-Unigene.fa.htm#gene108) | | 31 (0.13%) | | ko00430 | |
| 109 | [Monoterpenoid biosynthesis](../../../../E:%5C2011%E6%B5%8B%E5%BA%8F%E7%BB%93%E6%9E%9C%5Cbsi110817%5Cannotation%5CKEGG%5CAll-Unigene.fa.htm#gene109) | | 31 (0.13%) | | ko00902 | |
| 110 | [Other types of O-glycan biosynthesis](../../../../E:%5C2011%E6%B5%8B%E5%BA%8F%E7%BB%93%E6%9E%9C%5Cbsi110817%5Cannotation%5CKEGG%5CAll-Unigene.fa.htm#gene110) | | 31 (0.13%) | | ko00514 | |
| 111 | [Vitamin B6 metabolism](../../../../E:%5C2011%E6%B5%8B%E5%BA%8F%E7%BB%93%E6%9E%9C%5Cbsi110817%5Cannotation%5CKEGG%5CAll-Unigene.fa.htm#gene111) | | 30 (0.13%) | | ko00750 | |
| 112 | [Arachidonic acid metabolism](../../../../E:%5C2011%E6%B5%8B%E5%BA%8F%E7%BB%93%E6%9E%9C%5Cbsi110817%5Cannotation%5CKEGG%5CAll-Unigene.fa.htm#gene112) | | 27 (0.12%) | | ko00590 | |
| 113 | [Glycosphingolipid biosynthesis - globo series](../../../../E:%5C2011%E6%B5%8B%E5%BA%8F%E7%BB%93%E6%9E%9C%5Cbsi110817%5Cannotation%5CKEGG%5CAll-Unigene.fa.htm#gene113) | | 27 (0.12%) | | ko00603 | |
| 114 | [Synthesis and degradation of ketone bodies](../../../../E:%5C2011%E6%B5%8B%E5%BA%8F%E7%BB%93%E6%9E%9C%5Cbsi110817%5Cannotation%5CKEGG%5CAll-Unigene.fa.htm#gene114) | | 26 (0.11%) | | ko00072 | |
| 115 | [Brassinosteroid biosynthesis](../../../../E:%5C2011%E6%B5%8B%E5%BA%8F%E7%BB%93%E6%9E%9C%5Cbsi110817%5Cannotation%5CKEGG%5CAll-Unigene.fa.htm#gene115) | | 24 (0.1%) | | ko00905 | |
| 116 | [Sulfur relay system](../../../../E:%5C2011%E6%B5%8B%E5%BA%8F%E7%BB%93%E6%9E%9C%5Cbsi110817%5Cannotation%5CKEGG%5CAll-Unigene.fa.htm#gene116) | | 24 (0.1%) | | ko04122 | |
| 117 | [Indole alkaloid biosynthesis](../../../../E:%5C2011%E6%B5%8B%E5%BA%8F%E7%BB%93%E6%9E%9C%5Cbsi110817%5Cannotation%5CKEGG%5CAll-Unigene.fa.htm#gene117) | | 20 (0.09%) | | ko00901 | |
| 118 | [Thiamine metabolism](../../../../E:%5C2011%E6%B5%8B%E5%BA%8F%E7%BB%93%E6%9E%9C%5Cbsi110817%5Cannotation%5CKEGG%5CAll-Unigene.fa.htm#gene118) | | 16 (0.07%) | | ko00730 | |
| 119 | [Biotin metabolism](../../../../E:%5C2011%E6%B5%8B%E5%BA%8F%E7%BB%93%E6%9E%9C%5Cbsi110817%5Cannotation%5CKEGG%5CAll-Unigene.fa.htm#gene119) | | 12 (0.05%) | | ko00780 | |
| 120 | [C5-Branched dibasic acid metabolism](../../../../E:%5C2011%E6%B5%8B%E5%BA%8F%E7%BB%93%E6%9E%9C%5Cbsi110817%5Cannotation%5CKEGG%5CAll-Unigene.fa.htm#gene120) | | 11 (0.05%) | | ko00660 | |
| 121 | [Anthocyanin biosynthesis](../../../../E:%5C2011%E6%B5%8B%E5%BA%8F%E7%BB%93%E6%9E%9C%5Cbsi110817%5Cannotation%5CKEGG%5CAll-Unigene.fa.htm#gene121) | | 10 (0.04%) | | ko00942 | |
| 122 | [Lipoic acid metabolism](../../../../E:%5C2011%E6%B5%8B%E5%BA%8F%E7%BB%93%E6%9E%9C%5Cbsi110817%5Cannotation%5CKEGG%5CAll-Unigene.fa.htm#gene122) | | 9 (0.04%) | | ko00785 | |
| 123 | [Fatty acid elongation](../../../../E:%5C2011%E6%B5%8B%E5%BA%8F%E7%BB%93%E6%9E%9C%5Cbsi110817%5Cannotation%5CKEGG%5CAll-Unigene.fa.htm#gene123) | | 9 (0.04%) | | ko00062 | |
| 124 | [Caffeine metabolism](../../../../E:%5C2011%E6%B5%8B%E5%BA%8F%E7%BB%93%E6%9E%9C%5Cbsi110817%5Cannotation%5CKEGG%5CAll-Unigene.fa.htm#gene124) | | 8 (0.03%) | | ko00232 | |
| 125 | [Sesquiterpenoid biosynthesis](../../../../E:%5C2011%E6%B5%8B%E5%BA%8F%E7%BB%93%E6%9E%9C%5Cbsi110817%5Cannotation%5CKEGG%5CAll-Unigene.fa.htm#gene125) | | 4 (0.02%) | | ko00909 | |
| 126 | [Betalain biosynthesis](../../../../E:%5C2011%E6%B5%8B%E5%BA%8F%E7%BB%93%E6%9E%9C%5Cbsi110817%5Cannotation%5CKEGG%5CAll-Unigene.fa.htm#gene126) | | 2 (0.01%) | | ko00965 | |
