# Supplementary material for: De Novo Assembly of the Peanut (Arachis hypogaea L.) Seed Transcriptome Revealed Candidate Unigenes for Oil Accumulation Pathways
Source: PLoS One. 2013 Sep 10;8(9):e73767. doi: 10.1371/journal.pone.0073767 (PMC3769373; doi:10.1371/journal.pone.0073767)
Supplement: Table S12 — Fatty acid composition of the total lipid content in mature seeds. (DOC) [file pone.0073767.s013.doc]

Table S12. Fatty acid composition of total lipid in mature seeds

| lines | 16：0 | 18：0 | 18：1 | 18：2 | O/L | 18：3 | 20：0 | 20：1 | 22：0 | 24：0 | Oli content(%) |
| --- | --- | --- | --- | --- | --- | --- | --- | --- | --- | --- | --- |
| U12 | 10.55 | 4.06 | 50.29 | 29.11 | 1.70 | 0.25 | 1.55 | 0.81 | 2.11 | 1.26 | 44.98 |
| U606 | 10.85 | 4.24 | 40.75 | 38.05 | 1.07 | 0.15 | 1.80 | 0.72 | 2.49 | 0.95 | 59.15 |
